# The Buprenorphine Paradox: How Buprenorphine Triggers and Resolves Opioid Withdrawal

**DOI:** 10.1111/adb.70126

**Published:** 2026-03-09

**Authors:** Mehdi Haghdoost, Jennifer LaBranche, Matthew Roberts, Victor W. Li, Jane J. Kim, James S. H. Wong, Pouya Azar

**Affiliations:** ^1^ Aidos Innovation Sheridan Wyoming USA; ^2^ Complex Pain and Addiction Service, Department of Psychiatry Vancouver Coastal Health Vancouver Canada; ^3^ Department of Psychiatry, Faculty of Medicine University of British Columbia Vancouver Canada

**Keywords:** biased agonists, buprenorphine, mu‐opioid receptor, opioid use disorder, precipitated withdrawal

## Abstract

Buprenorphine (BUP) offers a therapeutic approach for opioid use disorder (OUD) due to its unique pharmacodynamic properties, primarily as a partial agonist with high affinity for the mu‐opioid receptor (MOR). BUP's partial agonism and ceiling effect on respiratory depression enhance its safety profile. However, BUP can induce precipitated withdrawal when administered after a full agonist, leading to severe withdrawal symptoms. This Perspective builds on prior work that has linked BUP's high‐affinity partial agonism to precipitated withdrawal and low‐dose induction strategies. We focus on how BUP's capacity to promote MOR externalization, together with its activity at the nociceptin opioid peptide (NOP) receptor, can help explain why it precipitates withdrawal when administered in the presence of full agonists yet relieves withdrawal once spontaneous withdrawal has begun. Understanding these mechanisms is critical for optimizing BUP protocols in OUD treatment and informs the potential development of new biased MOR agonists (i.e., ligands that preferentially activate specific signalling pathways) for addiction therapy.

## Graphical abstract

Buprenorphine can either precipitate or relieve opioid withdrawal depending on the timing of administration relative to full opioid agonist exposure. The article proposes that buprenorphine promotes mu‐opioid receptor (MOR) externalization and resensitization, creating a temporary signaling gap that explains precipitated withdrawal when full agonists are present, but withdrawal relief once spontaneous withdrawal has begun. Additional activation of the nociceptin (NOP) receptor may further contribute to its withdrawal‐modulating effects.

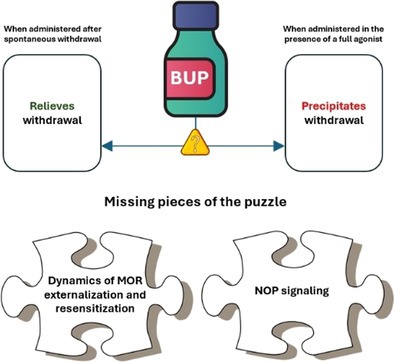

## 1 Introduction

The opioid epidemic remains a severe public health crisis globally, with a particular concentration in North America. In the United States alone, opioid overdoses led to over 80 000 deaths in 2023, reflecting a sustained level of mortality despite widespread efforts to curb the epidemic through policy, treatment and prevention strategies. This crisis has multifaceted impacts beyond mortality rates, as opioid use disorder (OUD) contributes to substantial social, economic and health burdens. Studies estimate the economic toll of the opioid crisis in the United States at over $1 trillion annually [1], accounting for healthcare costs, lost productivity, criminal justice involvement and other social costs. The crisis has also had a devastating impact on Canada, with over 8000 opioid overdose deaths and a rate of 21.1 per 100 000 population in 2023 [2].

Dependence, addiction and dose escalation due to tolerance significantly complicate opioid discontinuation and are key contributors to the development of OUD. With the rise of the opioid epidemic, the need arose for efficacious interventions to treat OUD and reduce the risk of death by overdose in individuals who continue to use. Buprenorphine (BUP) is a partial mu‐opioid receptor (MOR) agonist that was discovered in the 1970s as a safer alternative to heroin and morphine for pain management [3]. While it continues to be useful in the treatment of pain, its application for the treatment of OUD has grown in popularity. BUP confers several safety advantages as compared with the common alternative, methadone, given its reduced risk of QTc prolongation and ceiling effect on respiratory depression [3, 4]. Additionally, using a combination of BUP and naloxone limits the possibility of intravenous or intranasal abuse of the medication [4]. Studies of BUP's efficacy have suggested that it reduces overdose and all‐cause mortality, reduces high‐risk behaviours for transmission of HIV and other infectious diseases and improves quality of life ratings with maintenance treatment [5, 6]. However, a few factors have limited BUP's preference over methadone, including mixed evidence of lower retention rates, the requirement to experience withdrawal symptoms prior to standard BUP induction and the risk of precipitating withdrawal [4].

The use of BUP soon after the use of a full mu‐opioid agonist, such as fentanyl, can result in a precipitated withdrawal syndrome. Clinically, this presents with traditional opioid withdrawal symptoms, including anxiety, restlessness, nausea/vomiting, diarrhoea, aches, tremors, dilated pupils, rhinorrhoea and yawning. However, the onset of these symptoms in precipitated withdrawal is much less gradual, generally occurring within 2 h after the first dose of BUP and gradually improving over 6–24 h [7]. To prevent this from occurring, it is recommended that BUP be initiated after a period of at least 6–12 h of abstinence from short‐acting opioids and 24–72 h for long‐acting opioids [8, 9]. A newer strategy, first introduced as the Bernese method in 2016, uses low‐dose titrations of BUP over several days, which aims to reduce the risk of precipitated withdrawal without requiring prior cessation of full agonists and the onset of withdrawal. Since then, low‐dose inductions (previously known as microdosing and microinduction) have been widely applied across multiple clinical settings [10, 11]. Furthermore, rapid low‐dose inductions, spanning 1–3 days, have recently been developed and successfully implemented to initiate patients onto BUP and its extended‐release formulation [12, 13, 14, 15, 16]. Despite its general effectiveness, precipitated withdrawals using this approach can still occur in a small percentage of cases [17].

Understanding the mechanism of action of BUP in precipitated withdrawal is crucial, as it offers insights into the nuanced interactions between opioid receptors and withdrawal processes. By elucidating these mechanisms, researchers and clinicians can better predict the therapeutic effects of BUP and optimize dosing protocols to minimize withdrawal symptoms effectively. This understanding also opens pathways for refining treatment protocols for OUD and potentially informs the development of new therapeutic strategies for managing other substance use disorders or co‐occurring conditions.

## 2 Discussion

### 2.1 MOR Desensitization and Recovery

Desensitization of the MOR is a crucial mechanism underlying opioid tolerance and dependence [18, 19, 20], as it diminishes the efficacy of the drug over time, necessitating higher doses to achieve the same pharmacological effect. Desensitization involves a decrease in receptor responsiveness, which is primarily mediated by phosphorylation of the receptor by G‐protein–coupled receptor kinases (GRKs) [21]. This phosphorylation triggers the recruitment of β‐arrestin, which uncouples the receptors from G‐proteins and targets them for internalization through endocytosis [22]. The MOR can signal through two primary pathways: G‐protein–mediated signalling and β‐arrestin–mediated signalling. G‐protein signalling is traditionally believed to be associated with the analgesic and euphoric effects of opioids, while β‐arrestin signalling has been implicated in adverse effects such as tolerance [23]. However, recent studies have shown that β‐arrestin binding and receptor endocytosis are not necessary to produce desensitization of MOR [24]. In the absence of β‐arrestin, other nonarrestin mechanisms can efficiently desensitize the receptor, suggesting the existence of alternative pathways for MOR regulation [25]. Agonists of opioid receptors, including MOR, exhibit varying efficacies in activating the G‐protein and β‐arrestin signalling pathways. This phenomenon, referred to as biased agonism [26], allows certain ligands to preferentially activate one pathway over the other, thereby influencing the development of tolerance, efficacy and side effect profiles.

Recovery from MOR desensitization is typically rapid, allowing receptors to regain functionality after the cessation of opioid exposure [24]. However, chronic opioid treatment can significantly impair this recovery process. The extent and nature of recovery depend on the specific opioid used during chronic treatment. For instance, chronic morphine treatment has been shown to inhibit receptor recycling, which hampers the ability of the receptors to return to the cell surface and participate in signalling once again [27, 28]. This inhibition leads to a prolonged state of desensitization and reduced receptor availability. A similar decrease in MOR availability has been observed in vivo with chronic administration of heroin [29, 30] and is expected from chronic dosing of fentanyl. Conversely, chronic treatment with methadone does not exhibit the same inhibitory effect on receptor recycling, allowing for more efficient recovery of MOR functionality [28].

### 2.2 Interaction of BUP With MOR

BUP exhibits a significantly higher binding affinity to the MOR (K*i* ~ 0.2 nM) compared with that of potent full agonists like morphine and fentanyl (K*i* ~ 1 nM) [31]. Consequently, BUP can readily displace these opioids and, at sufficient concentrations, can also replace full MOR agonists with even higher affinities. However, the extent of receptor replacement depends on the relative binding affinities and concentrations of the competing ligands. For very high‐affinity opioids such as sufentanil, proportionally higher doses of BUP are required to achieve substantial receptor displacement. As a partial agonist, BUP does not fully activate the receptor and acts as a biased agonist of MOR [31]. Among the two major intracellular pathways activated upon binding to MOR, BUP exhibits a significantly higher affinity for the G‐protein pathway compared with the arrestin pathway [32]. In contrast, full agonists such as morphine and fentanyl demonstrate significantly less signalling bias compared with BUP [33]. The G‐protein bias of BUP results in selective activation of specific opioid effects rather than a uniform response across all opioid‐mediated endpoints. For example, BUP demonstrates full clinical analgesic efficacy [34] while exhibiting a markedly lower risk of respiratory depression compared with unbiased opioids such as fentanyl [35].

One important aspect of BUP pharmacology is its ability to prevent MOR desensitization and internalization, typically caused by potent opioid agonists [36]. In vitro studies further suggest that BUP can act as an externalization ligand of MOR. While etorphine, fentanyl and morphine cause 50%, 35% and 10% receptor internalization, respectively, after 1 h of treatment, BUP not only fails to promote internalization but also increases the number of receptors on the cell surface (i.e., externalization) under similar conditions by 10% [37]. The data also suggest that BUP can block agonist‐induced MOR internalization with an EC_50_ = 13 nM. Although a partial agonist of the receptor, BUP's ability to cause receptor externalization is astonishingly similar to that of MOR antagonists like naloxone (EC_50_ = 28 nM).

### 2.3 BUP‐Induced Precipitation of Withdrawal

**FIGURE 1:**
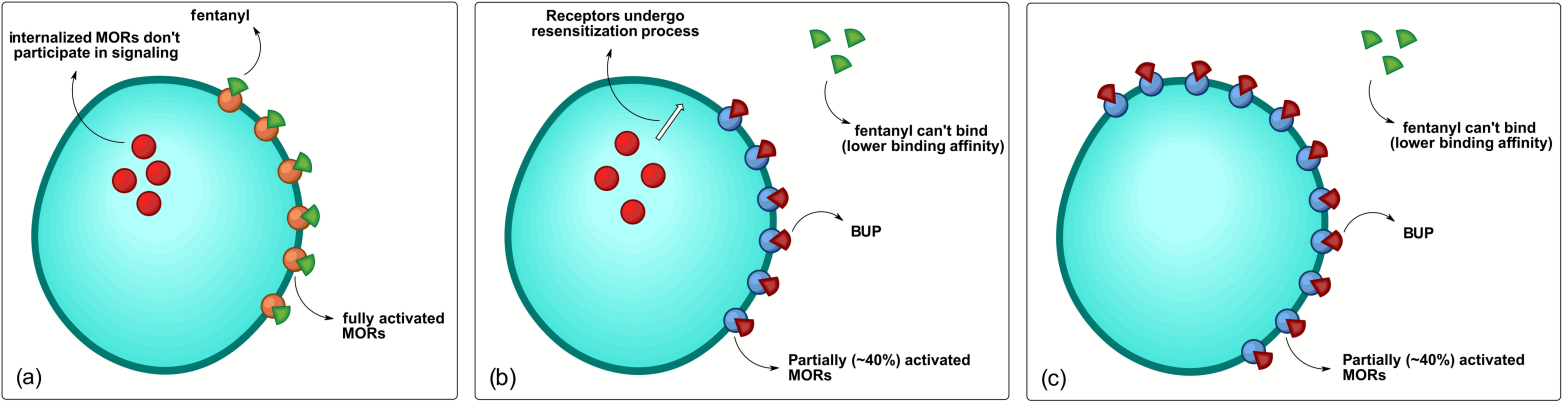
State of MORs (a) when a full agonist such as fentanyl is onboard, (b) after the introduction of BUP while internalized receptors are in the resensitization process (precipitated withdrawal) and (c) after full receptor recovery (alleviation of withdrawal).

Prolonged full agonist opioid exposure causes a significant number of MORs to become desensitized and internalized, rendering them incapable of participating in signalling (Figure 1a). The longer the exposure to opioids, the slower the recovery of these receptors from desensitization. As a high‐affinity ligand, BUP outcompetes other opioids in binding to the MOR. However, due to its partial agonism with a ceiling effect and the fact that a substantial proportion of MORs are internalized and thus unable to participate in signalling, BUP binding leads to a rapid decline in MOR‐mediated signalling, triggering the onset of withdrawal symptoms. This mechanistic framework largely aligns with that proposed by De Aquino et al., who used BUP partial agonism to explain both precipitated withdrawal and the rationale for low‐dose and microinduction protocols [38]. However, the proposed mechanism can be further extended to better align with clinical observations related to BUP‐induced withdrawal precipitation. For instance, when BUP is administered immediately after a full agonist such as fentanyl, it triggers the onset of rapid withdrawal symptoms. In contrast, if a patient enters spontaneous withdrawal following the discontinuation of a full agonist, BUP does not worsen withdrawal symptoms but instead can effectively address the patient's opioid requirements.

Explicitly incorporating the timing of BUP‐induced MOR externalization and resensitization helps reconcile why the same drug can both trigger and resolve withdrawal under different clinical conditions. Acting as a biased agonist of MOR (lack of activity in arrestin pathway), BUP is believed to achieve the MOR externalization by shifting the receptor signalling to the G‐protein pathway exclusively. When a full agonist is onboard, BUP administration prevents further receptor internalization and promotes the externalization and recovery of desensitized receptors. In vitro data indicate that receptor externalization occurs relatively rapidly within the first hour of BUP exposure. Extending the exposure to 18 h results in a modest but additional increase in surface receptor expression compared with the 1‐h treatment [37]. The timeline for BUP‐induced MOR recovery in the clinical setting has not been thoroughly explored. However, it is expected that this transition is not immediate but contains a gap between the timings of BUP administration and MOR externalization and recovery (Figure 1b). This gap is significant in understanding BUP‐induced withdrawal as it reflects the time required for internalized receptors to regain sensitivity. It is suspected that during this time, shortly after BUP administration, is when symptoms of precipitated withdrawal are observed. Once resensitized, these receptors can be reactivated by BUP (Figure 1c), leading to an increase in MOR signalling, subsequently alleviating withdrawal symptoms and meeting the patient's opioid requirement.

According to the proposed mechanism, BUP administration can avoid precipitating withdrawal if internalized MOR are resensitized prior to BUP initiation. This resensitization can be achieved either by discontinuing full opioid agonists (spontaneous withdrawal) or by administering a MOR antagonist, such as naloxone [39, 40], to accelerate receptor resensitization. This mechanism explains the key difference between spontaneous withdrawal and BUP‐induced precipitated withdrawal. During spontaneous withdrawal, MOR externalization occurs gradually as the full agonist opioid and its metabolites are cleared from the system. The decline in receptor occupancy and the subsequent resensitization of internalized MORs follow a relatively slow, time‐dependent process. In contrast, when BUP is introduced in the presence of a full agonist, it acts as a pharmacologic externalization agent. Because of its high affinity and partial agonism, BUP rapidly displaces the full agonist from MORs while simultaneously promoting receptor externalization. This creates an abrupt mismatch between receptor activation and receptor availability, leading to the rapid onset of precipitated withdrawal.

This mechanistic framework also clarifies why BUP low‐dose inductions, a strategy involving small and incremental doses of BUP titration [12, 15, 41], may mitigate the likelihood of precipitated withdrawal. By gradually displacing residual opioid agonists while simultaneously promoting MOR externalization and functional recovery, low‐dose inductions align BUP's ascending receptor occupancy with the progressive restoration of responsive MORs. This approach bridges the critical gap in MOR signalling, ensuring that BUP's partial activation of MOR is synchronized with receptor availability.

### 2.4 Interaction of BUP With Nociceptin Opioid Peptide (NOP)

Whereas prior mechanistic accounts have largely focused on MOR regulation, we also consider BUP's actions at the opioid peptide receptor (NOP) and how this antiopioid signalling may interact with MOR dynamics to shape the clinical expression of withdrawal. NOP, a member of the opioid G‐protein–coupled receptor family, is activated by the 17‐amino acid neuropeptide nociception [42]. Activation of NOP indirectly inhibits the MOR and kappa‐opioid receptors (KORs), leading to antiopioid effects in certain tissues [43]. BUP is one of the few MOR agonists with significant affinity for NOP, albeit with lower affinity compared with its binding to MOR and KOR [31]. Despite this, the role of NOP activation by BUP in precipitated withdrawal has been largely overlooked. Although NOP is structurally related to opioid receptors, its activation is not associated with reinforcing effects. Instead, NOP signalling has been shown to suppress opioid‐mediated rewards in both rodents and non‐human primates [44]. Consequently, bifunctional NOP/MOR agonists like BUP are expected to exhibit lower rewarding properties compared with selective MOR agonists [45]. Additionally, BUP's agonism at NOP compromises its own pain‐relieving effects [46]. As a result, when a full MOR agonist such as fentanyl is present, BUP administration not only causes MOR receptor externalization but also can negatively affect the analgesic and rewarding effects of the full agonist by acting as an agonist of NOP.

The extent to which NOP influences BUP‐induced precipitated withdrawal is determined by its relative affinities and functional activities at both the NOP and MOR receptors [47]. However, in vitro affinity values for most opioids, including BUP, vary widely in the literature, leading to inconsistencies. For example, Wnendt et al. first reported an IC_50_ of 8.4 nM for BUP's agonist activity at NOP [48], whereas most studies indicate a K*i* and IC_50_ of 0.2–1.5 nM for its partial agonist activity at MOR [49]. Despite its lower affinity, BUP binding to NOP leads to greater receptor stimulation (~60%) than its binding to MOR (~38%), suggesting that BUP is more efficacious at NOP [50]. Adding to this complexity, BUP metabolites, including BUP‐3‐glucuronide and norbuprenorphine‐3‐glucuronide, exhibit significant activity at NOP, which may differ from that of the parent compound [51]. Further research on the activity of BUP and its metabolites at opioid and opioid‐like receptors, in both preclinical and clinical settings, is necessary to fully elucidate the role of NOP in modulating the overall biological effects of BUP.

## 3 Conclusion

BUP occupies a unique position in OUD treatment. In this Perspective, we build on the previously reviewed BUP MOR‐based framework in two ways: (1) We more explicitly temporalize the dynamics of MOR externalization and resensitization during transitions from full agonists to BUP, and (2) we integrate NOP signalling into the same receptor‐level model in order to better account for the clinical observations. Together, these additions may help clarify why BUP given immediately after a full agonist can precipitate withdrawal, whereas BUP introduced once spontaneous withdrawal has begun does not worsen symptoms and instead relieves opioid craving. The same mechanistic framework also supports the rationale for low‐dose and microinduction protocols, which appear to reduce the risk of severe precipitated withdrawal. The discussed mechanism emphasizes the need for precision in clinical protocols, such as tailoring dosing schedules to the receptor dynamics of individual patients and employing strategies to minimize the risk of precipitated withdrawal and achieve therapeutic dosing. A key criterion for successful OUD treatment, based on the suggested mechanism, is to maximize MOR externalization while minimizing withdrawal symptoms in the shortest time frame possible.

On a broader scale, the mechanistic knowledge of MOR desensitization and recovery provides a framework for developing novel therapeutic agents. Future biased agonists could be designed to exploit specific signalling pathways, optimizing efficacy while minimizing adverse effects such as respiratory depression or withdrawal symptoms. Additionally, integrating these molecular insights into patient management strategies, such as monitoring receptor recovery timelines, can enhance the effectiveness of OUD treatments and improve patient outcomes. Future research should aim to validate the proposed mechanism experimentally. One of the most direct and informative approaches would be pharmacological brain imaging to assess changes in MOR availability at different stages of OUD treatment with BUP. Positron emission tomography (PET) using radiolabelled BUP could provide real‐time insights. Such studies would help determine the relationship between receptor availability and withdrawal resolution, offering an evidence‐based framework for optimizing induction protocols and guiding the design of next‐generation MOR agonists with improved safety and tolerability profiles.

## Author Contributions

All the authors meet the ICMJE authorship criteria and have made significant and equal contributions to this manuscript. All authors have read and agreed to the published version of the manuscript.

## Funding

This work was supported by VGH and UBC Hospital Foundation.

## Conflicts of Interest

M.H., M.R. and P.A. are affiliated with Aidos Innovation, a non‐profit organization focused on developing, evaluating and implementing disorder treatments for substance use disorders, mental health disorders and pain. P.A. was a consultant on Indivior‐led buprenorphine extended‐release studies (terminated in 2023) and receives honoraria for presentations organized by Indivior.

## Data Availability

Data sharing is not applicable to this article as no datasets were generated or analysed during the current study.
